# Severe Lower Gastrointestinal Bleeding Leading to Bilateral Non-arteritic Anterior Ischemic Optic Neuropathy: A Rare Systemic Complication

**DOI:** 10.7759/cureus.83119

**Published:** 2025-04-28

**Authors:** John K Appiah, Emmanuel K Asiedu, Edward A Danso

**Affiliations:** 1 Internal Medicine, Geisinger Health System, Wilkes-Barre, USA; 2 Internal Medicine, Mother and Child Hospital, Accra, GHA; 3 Surgery, Korle-Bu Teaching Hospital, Accra, GHA

**Keywords:** acute blood loss anemia, anemia, carotid artery disease, diverticular bleeding, lgib, non-arteritic anterior ischemic optic neuropathy (naion)

## Abstract

Lower gastrointestinal bleeding (LGIB) is a common clinical emergency; however, systemic complications such as bilateral non-arteritic anterior ischemic optic neuropathy (NAION) are exceedingly rare. We report the case of a 78-year-old male with a history of hypertension and hyperlipidemia who developed progressive bilateral vision loss following hospitalization for severe LGIB. His hemoglobin nadir dropped to 6.9 g/dL from a baseline of 11 g/dL, prompting transfusion with one unit of packed red blood cells. Arteritic causes were ruled out through appropriate workup. Imaging demonstrated significant carotid and vertebrobasilar atherosclerotic disease, and ophthalmologic evaluation confirmed bilateral NAION. This case highlights the potential for ischemic optic neuropathy in the setting of acute anemia and underlying vascular insufficiency. Clinicians should maintain a high index of suspicion for ischemic complications following severe gastrointestinal bleeding, particularly in high-risk patients.

## Introduction

Lower gastrointestinal bleeding (LGIB) is a frequent cause of hospital admissions in elderly patients, most commonly due to diverticular bleeding [1]. This usually carries localized risks, but systemic complications, especially involving end-organ ischemia, are rarely reported [1,2]. In select cases, profound anemia and hypoperfusion may trigger ischemic complications, particularly in patients with compromised vascular reserve [2,3].

Non-arteritic anterior ischemic optic neuropathy (NAION) is the most common optic neuropathy in adults over 50 and is typically unilateral [4]. Bilateral simultaneous NAION is rare and often associated with hypotension, acute blood loss, or anemia [4-6]. In elderly patients, giant-cell arteritis (GCA) should also be considered; the American College of Rheumatology criteria remain widely used for diagnosis [7]. We report a rare instance of bilateral NAION in the setting of LGIB-induced anemia and significant cerebrovascular disease.

## Case presentation

A 78-year-old male with a medical history of hypertension and hyperlipidemia presented with bilateral vision loss of two weeks’ duration. He reported that symptoms began a few days after discharge from a recent hospitalization for gastrointestinal bleeding, initially as blurring in both eyes, which progressively worsened to near-total blindness. Despite early symptom onset, he delayed seeking medical attention. He denied associated pain, headache, jaw claudication, or scalp tenderness.

Two weeks prior, the patient had been hospitalized for profuse rectal bleeding lasting for four days. On admission, his hemoglobin was 6.9 g/dL, a significant decline from his baseline of 11 g/dL. He received one unit of packed red blood cells. Colonoscopy revealed no active bleeding or stigmata, but noted severe sigmoid diverticulosis. A self-limited diverticular bleed was suspected, though not definitively confirmed. He was stabilized and discharged home.

**Figure 1:**
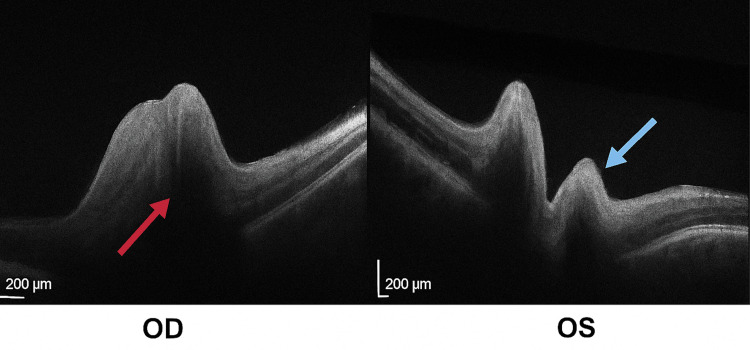
Optical coherence tomography (OCT) findings of both eyes. OCT B-scan images of both eyes demonstrating optic disc edema in non-arteritic anterior ischemic optic neuropathy. The red arrow indicates optic disc edema in the right eye (OD), while the blue arrow highlights elevation and swelling of the optic nerve head in the left eye (OS). Scale bars: 200 µm.

At the time of ophthalmologic evaluation, visual acuity was 20/200 in the right eye (OD) and 20/400 in the left eye (OS), with intraocular pressure of 16 mmHg in both eyes (OU). Optical coherence tomography revealed bilateral optic disc edema (Figure 1).

Inflammatory marker levels (Table 1), along with temporal artery biopsy findings of intimal hyperplasia and calcified atherosclerotic plaque, consistent with chronic hypertension changes, effectively ruled out arteritic etiologies such as GCA.

**Table 1:** Inflammatory marker levels. Inflammatory marker levels at the time of presentation. The C-reactive protein was within the normal range at <3 mg/L. The erythrocyte sedimentation rate was mildly elevated at 23 mm/hour. These findings, when considered with a non-diagnostic temporal artery biopsy, helped rule out arteritic etiologies such as giant-cell arteritis.

Test	Reference range	Result
C-reactive protein	≤5 mg/L	<3 mg/L
Erythrocyte sedimentation rate	≤20 mm/hour	23 mm/hour

CT angiography of the neck showed severe atherosclerosis with high-grade stenoses in both carotid arteries: right common carotid artery origin (~70%) and distal segments (up to 80-90%), and left common carotid artery occlusion with reconstitution and 80% left internal.

## Discussion

Bilateral NAION is a rare but devastating complication typically associated with systemic hypoperfusion, often in the context of shock, profound anemia, or hypotension, particularly among individuals with underlying vascular disease or compromised cerebral circulation [2,5,6,8,9]. In this case, significant LGIB led to acute anemia and likely contributed to optic nerve head hypoperfusion. The patient’s preexisting severe bilateral carotid and vertebrobasilar disease further impaired cerebral autoregulation and left the optic nerves especially vulnerable during the hypoperfusion episode [5,9].

NAION is caused by infarction of the optic nerve head, usually from impaired circulation in the posterior ciliary arteries, which are end arteries lacking collateral supply. Classic risk factors include small optic discs (“disc-at-risk”); systemic vascular conditions such as hypertension, diabetes, and hyperlipidemia; and anatomic variations such as crowded optic nerves [3,4]. However, when bilateral NAION occurs simultaneously or in close succession, systemic triggers must be suspected. Hayreh [4] highlighted anemia and systemic hypotension as significant contributors in such cases, particularly among elderly patients with reduced vascular reserve [2,5]. The extent of the optic nerve damage correlates not only with the duration and severity of hypotension or anemia but also with preexisting cerebrovascular insufficiency, as in our patient [4,9].

While diverticular hemorrhage is typically self-limited, it may lead to substantial acute blood loss, especially in elderly or anticoagulated patients [1]. In this population, the risk of ischemic end-organ complications, including cerebral or ocular ischemia, is heightened, particularly in the presence of significant atherosclerotic disease or impaired collateral flow [1,6]. A growing body of literature now recognizes the optic nerve as one such end organ susceptible to ischemic damage during episodes of systemic compromise. Hayreh reported a similar case of bilateral NAION following gastrointestinal hemorrhage, emphasizing the need for rapid hemodynamic stabilization and neurologic assessment in such contexts [6].

While the patient was found to have severe bilateral carotid artery disease, no acute intervention was pursued during hospitalization due to his delayed presentation and established bilateral vision loss. He was referred to vascular surgery for outpatient evaluation, and medical management was optimized with antiplatelet therapy and statin intensification.

Furthermore, this case underscores the need for heightened clinical suspicion and multidisciplinary collaboration. Gastrointestinal specialists managing acute LGIB in patients with advanced vascular disease should remain vigilant for subtle neurologic complaints such as vision loss or transient visual obscurations. Collaboration with neurology, ophthalmology, and vascular surgery is essential to expedite diagnosis and optimize management. Although colonoscopy is generally safe, complications such as significant postprocedural bleeding can occur and should be factored into risk stratification, especially in patients with predisposing systemic or vascular comorbidities [8]. Moreover, vision loss in elderly patients has been shown to significantly impact quality of life and is associated with increased morbidity and mortality [10]. Vascular risk factors are increasingly recognized as contributors to ischemic optic neuropathies, highlighting the importance of systemic optimization in at-risk populations [11].

## Conclusions

Severe LGIB can rarely lead to bilateral NAION due to ischemic complications. The risk is significantly increased in patients with underlying carotid or vertebrobasilar disease, particularly in the setting of anemia or systemic hypoperfusion. Gastrointestinal physicians should be aware that vision loss may present as a delayed complication following significant bleeding events. In such scenarios, prompt multidisciplinary involvement, including neurology, ophthalmology, and vascular surgery, is crucial for optimal patient management.
